# Strain Effect on the Electronic and Optical Properties of CdSe Nanowires

**DOI:** 10.1186/s11671-017-1952-9

**Published:** 2017-03-09

**Authors:** Hao Huan, Li Chen, Xiang Ye

**Affiliations:** 1 Department of Physics, Shanghai Normal University, Shanghai, 200234 People’s Republic of China; 2 School of Arts and Sciences, MCPHS University, Boston, MA 02115 USA

**Keywords:** Strain, Cadmium selenide, Nanowire, Electronic properties, Optical properties

## Abstract

First-principles density functional theory (DFT) simulations were carried out to study the strain dependence on the electronic and optical properties of cadmium selenide (CdSe) nanowires (NWs). The band structures, effective masses of electron and holes, dielectric properties, and other optical properties (such as extinction coefficient, optical reflectivity, and absorption coefficient) were calculated under both compressive and tensile uniaxial strains. Size-dependence was also discussed by comparing results among CdSe wires with various diameters. Simulation results show that an interesting band-switch behavior occurs at the valence bands regardless of size. The cause and the consequences of such band-switch behavior were also studied. Further strain dependence on corresponding electronic and optical properties were examined as well. Our results provide insights to possible mechanical tuning via strain on the electronic and optical properties of CdSe NWs.

## Background

*C*admium selenide (CdSe) nanostructures have attracted much attention due to their potential applications in micro- and nano-optoelectronics [1–5]. Recently, a great variety of CdSe nanostructures including nanowires (NWs) [6], nanospheres [7], nanorods [8], nanosheets [9], and nanocrystals [10] have been successfully synthesized. The structural, electronic, and optical properties of various CdSe nanostructures were also widely studied [1, 11–14]. Among all CdSe nanostructures, CdSe NWs are by far the most attractive to researchers and scientists due to their unique opto-electrical properties, high length-diameter aspect ratio, and high surface-to-volume ratio. Especially CdSe NWs has been shown as good field-effect transistor [15] similar to carbon nanotube bundles [16]. Experimentally, CdSe NWs have been successfully synthesized by various methods [6, 17–19], such as *γ*-irradiation, electrochemistry, and solution–liquid–solid (SLS). Depending on the synthesis conditions, researchers were able to get two structures of CdSe nanowires: the zinc blende (ZB) structure and wurtzite (WZ) structure [6, 20–23]. Most WZ wires grew along [0001] crystallographic direction [23, 24]. Since then, many efforts have been put into the study of the electronic, thermal, and optical properties of CdSe NWs as well [25, 26]. However, semiconductor NWs, as basic units of microelectronic and optoelectronic nanodevices, often work under the existence of strain. Therefore, it is essential to also study the influence of strain on various structural, electronic, and optical properties of NWs. Indeed, strain effect has been widely studied on many nano-systems such as GaAs [27] and ZnO [28]. Interesting results were published to enrich our understanding. In 2013, Peng et al. found that GaAs nanowires undergo a direct–indirect bandgap transition under compressive strain. The studies of Lu group showed that the applied strain also affects the dielectric function peaks of ZnO nanowires. However, very limited information is available on how strain affects the electronic and optical properties of CdSe NWs. Previous studies have shown that first-principles DFT calculations well performs when it comes to the simulations of CdSe nano-systems [26, 29, 30]. Thus, in order to achieve a better understanding of the stain effect on CdSe NWs, we have carried out DFT calculations to investigate the effect of strain on the band structures and optical properties of CdSe NWs of various diameters.

## Methods

The nanowire structures were optimized using Atomistix Toolkit (ATK) package [31–33] under generalized gradient approximation (GGA). Perdew–Burke–Ernzerhof exchange-correlation functional [34] and L-BFGS optimizer [35] were used. The convergence criteria were set to be 1 × 10^−5^ eV/supercell for energy and 0.05 eV/Å for forces. The energy cutoff was set to be 75 Ha. The electronic and optical properties were calculated via Meta-GGA (MGGA) method with Tran and Blaha (TB09) functional [36] within the ATK package. Troullier–Martins [37] pseudopotentials were used, and the relativistic core corrections were also included. In both GGA and MGGA calculations, the Brillouin zone of CdSe NW is sampled using a (1 × 1 × 21) Monkhorst-Pack [38] special k-point mesh, which yields well-converged results. A vacuum region of at least 15 Å is used to avoid possible interactions between adjacent NWs that may be introduced due to periodic boundary conditions.

**Fig. 1:**
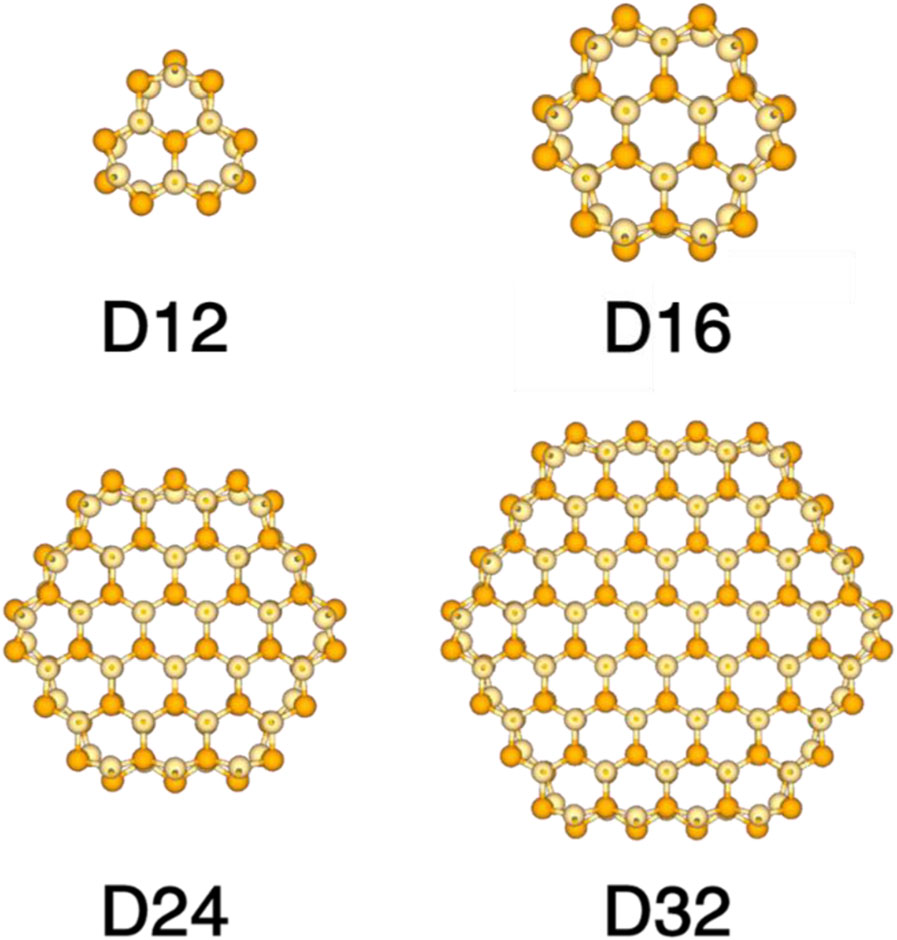
Cross-sectional structures of CdSe nanowires with diameters of 12.15 Å (labeled as D12), 15.82 Å (labeled as D16), 23.95 Å (D24), and 32.13 Å (D32)

Based on experimental results [39–41], we generated CdSe NWs along [0001] direction with $\left\{10\overline{1}0\right\}$ sidewall facets. Figure 1 gives the cross-sectional structures of four CdSe NWs studied in this paper. The radius of the NWs with a hexagonal cross-section is defined as an average of two radial dimensions: center to corner and center to edge distances. Their diameters are 12.15, 15.82, 23.95, and 32.13 Å, labeled as D12, D16, D24, and D32, respectively. Both the atom coordinates and the lattice constants of NWs are allowed to fully relax under all strain types and strengths. The amount of strain is described as *η* = (*d* − *d*_0_)/*d*_0_, where *d*_0_ is the original optimized lattice constant for CdSe NWs without strain, and *d* is the new lattice constant under strain. Positive values of *η* refer to tensile strain while negative values correspond to compressive strain. The amount of strain discussed in this paper ranges from *η* = −0.06 (6%, compressive) to *η* = +0.06 (6%, tensile) with an increment of 0.02 (2%). The effective masses of electron and hole are calculated as *m* ^∗^ = *ℏ* ^2^/(*d* ^2^ *E*/*dk* ^2^) at the conduction band minimum (CBM) and valence band maximum (VBM).

## Results and Discussion

**Fig. 2:**
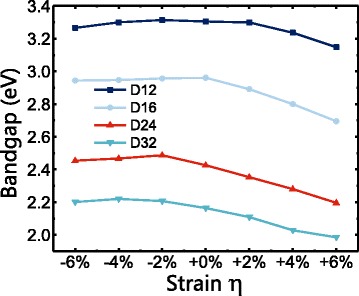
Bandgap of CdSe NWs under strain for CdSe NWs with various diameters

Figure 2 gives the bandgap of CdSe NWs under strain. Due to quantum confinement effect, greater gaps are found in NWs with greater diameters. For NWs with smaller diameters (i.e., D12 and D16), very little change in bandgap is found when the NW is under compressive strain. However, as tensile strain is applied, we see a much obvious decrease in the values of bandgap. For NWs with larger diameters (D24 and D32), bandgap starts to decrease noticeably at a strain level of −2%. It is worth pointing out that for D24, bandgap actually slightly increases under compressive strain between −6 and −2% before it decreases.

**Fig. 3:**
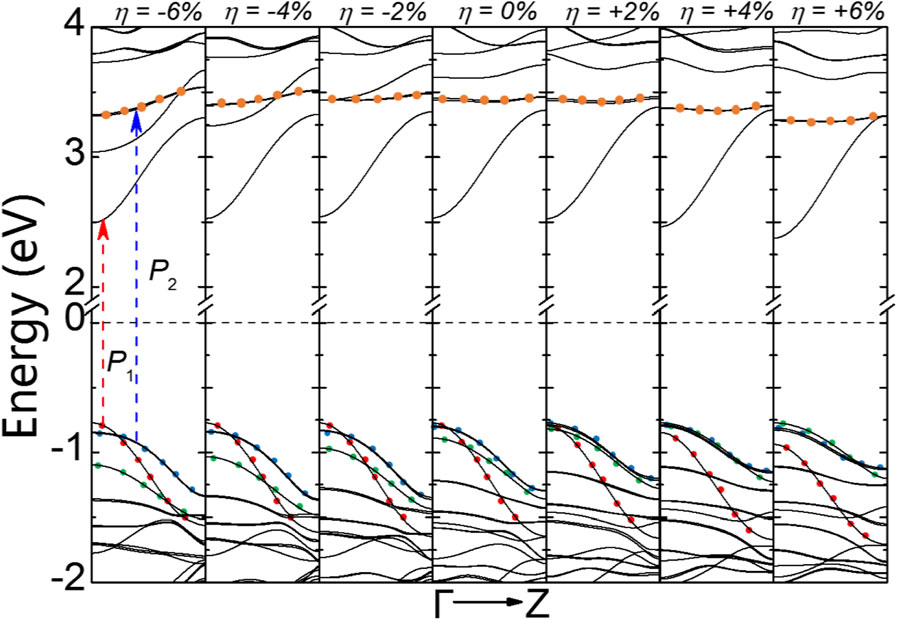
Band structures of D12 under compressive and tensile strain of various strength

**Fig. 4:**
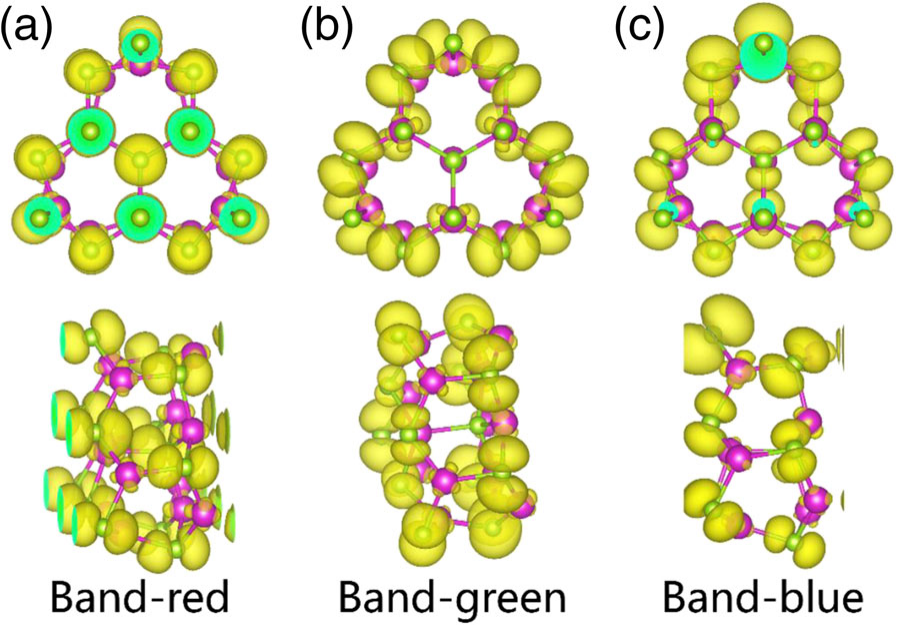
(*Upper*) top views and (*bottom*) side views of iso-value charge density contours of **a** the red band, **b** the green band, and **c** the blue band for D12 wire

Figure 3 gives the band structure of D12 as strain varies from −6 to +6%. Both VBM and CBM are located at Γ point, regardless of the amount of strain applied onto the system. A direct bandgap of 3.30 eV is found when the wire is fully relaxed under zero strain. The gap reduces to 3.27 eV under compressive strain of −6% and 3.15 eV under tensile strain of +6%. It is interesting to point out that the location of VBM almost does not change under different strain types and amount. However, CBM, on the other hand, moves down towards the Fermi level under tensile strain, which consequently reduces the bandgap of the nanowire. To further investigate how strain affects the electronic band structure of NWs, we traced the location of top three valence bands (colored as red, blue, and green in Fig. 3). As strain increases, the red band gradually sinks to the lower energy region, and the green band rises, while the blue band stays relatively at the same place. Under compressive and zero strain, the energy of red band at Γ point is the highest among the three and hence gives VBM. However, as it gradually moves down, the blue band becomes the highest and contributes to VBM at tensile strain of +2%. As the strain further increases to +6%, the rising green band exceeds the blue band and defines VBM. It is the shifts of these very bands that give rises to the change in the bandgap. To gain an insightful understanding on the band shifting, we have plotted the iso-value contours of charge density in Fig. 4 for these three bands. From Fig. 4a, we see that the red band is mainly contributed from bonds that are along parallel directions of strain. As compressive strain is applied, the amount of orbital overlap of these bonds increases, which results in a higher energy. On the other hand, from Fig. 4b, we see that the green band is mainly contributed from bonds that are perpendicular to strain direction. These bonds actually become more stable under compressive strain and hence lead to lower energy. Finally from Fig. 4c, the bonds contributing to the blue band are neither along nor perpendicular to the strain direction, which makes the blue band less sensitive to strain, as we have seen in Fig. 3.

**Fig. 5:**
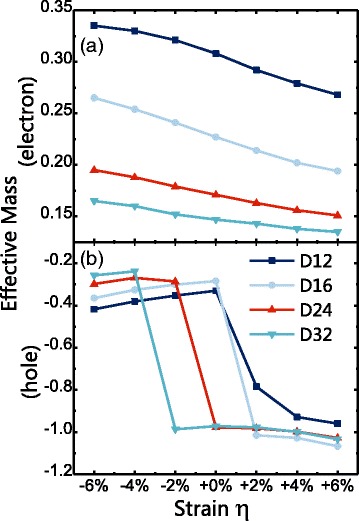
The effective mass of **a** electrons and **b** holes of CdSe NWs under strain

We plot the effective masses of electrons and holes under various strain strength in Fig. 5. The effective mass of electrons decreases as strain increases for all nanowires, as shown in Fig. 5a. It is interesting to point out that, compared to tensile strain, the effective mass of electrons reduces more under compressive strain, especially for wires with greater diameters. This is due to the fact that the curvature of CBM at the Γ point is more sensitive to compressive strain. While the effective mass of electrons reduces slowly and almost linearly under increasing strain, the effective mass of the holes has a more drastic drop as strain increases. As shown in Fig. 5b, the effective mass of holes for D12 initially has a slight increase from −0.418 to −0.330 as *η* increases from −6 to 0%. However, it then shows a sudden decrease to −0.785 at +2% and −0.936 at +4%, after which the change becomes slow and steady again. The same behavior can be found in all other wires. In fact, such a sudden drop is a direct result from band shifting as discussed in earlier sections. For D12, the blue band (in Fig. 3) replaces the red band at +2% and starts to define VBM. As the strain further increases, the green band rises and overwrites the blue band to give VBM. Indeed, after the green band becomes the top band, the effective mass of holes does not change drastically any more. This switch (in terms of which band defines VB) is shown to be more weak vs. strain in wires with greater diameters. In fact, the greater the diameter, the later the switch can be found. As strain *η* decreases, the red band sinks down more slowly in wires with larger diameters due to smaller quantum confinement effect [42, 43].

**Fig. 6:**
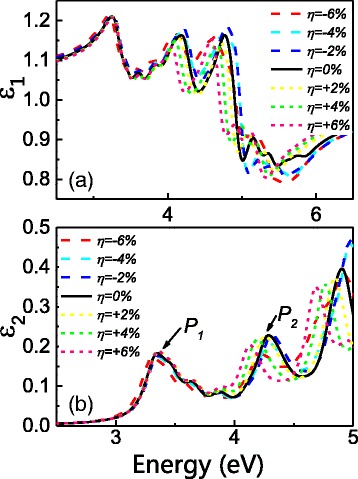
The **a** real and **b** imaginary part of the complex dielectric function *ε* = *ε*_1_ + *ε*_2_ for D12 wire under strain. The corresponding interband transitions of P1 and P2 are labeled in Fig. 3

It is also worth looking at how strain affects the interband optical properties of the wires as a consequence of its effect on band structures. Thus, we carried out calculations on the complex dielectric function *ε* = *ε*_1_(*ω*) + *iε*_2_(*ω*). Figure 6 gives the *ε*_1_ and *ε*_2_ as functions of energy for D12. The two peaks of *ε*_2_ are located at 3.3 eV (P1) and 4.3 eV (P2) under zero strain, as shown in Fig. 6b. P1 corresponds to an interband transition between the lowest conduction band and the highest valence band at Γ point, while P2 is associated with a transition from the blue band to the orange band at Γ point (as shown in Fig. 3). From Fig. 6b, we see that P1 almost does not move despite a small redshift at −6%. This is in consistent with our findings on bandgap in earlier sections. P2, on the other hand, has a more obvious redshift under tensile strain, which is due to the fact that the orange band (in Fig. 3) floats down under tensile strain.

**Fig. 7:**
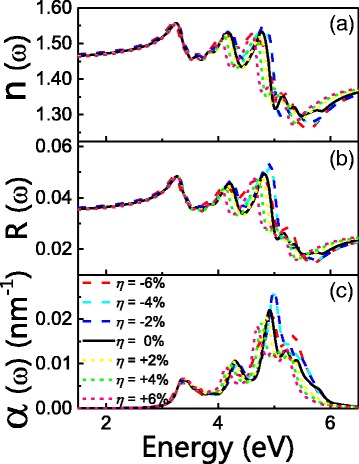
Strain effect on **a** refractive index *n*, **b** reflectivity *R*, and **c** absorption constant *α* of D12 wire

The index of refraction *n*, the extinction coefficient *k*, the optical reflectivity *R*, and the absorption coefficient *α* can all be calculated from *ε*_1_ and *ε*_2_. Detailed calculation procedures can be found in our previous works. In Fig. 7, we plot the calculated values of *n*, *R* and *α* of D12. The maximum value of *n* is found to be 1.56 at 3.25 eV at zero strain. This value does not change too much under other stain strength and/or types, as shown in Fig. 7a. Similarly in Fig. 7b, the reflectivity *R* almost stays the same in low energy region less than 4 eV. This indicates that the transparency of CdSe wires will not be strongly affected by strain. For higher energies (>4 eV), *R* generally decrease with increasing strain. The first two peaks in absorption coefficient *α* (Fig. 7c) has trends in consistent with the results of *ε*_2_. The highest also undergoes a redshift from 4.99 to 4.69 under increasing strain.

**Fig. 8:**
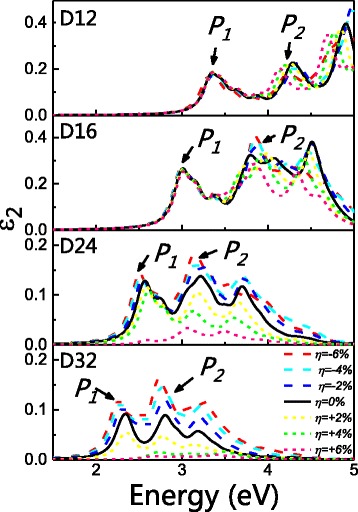
Strain effect on dielectric property *ε*_2_ for all wires

Figure 8 gives the calculated *ε*_2_ for all wires. Due to quantum confinement effect, all peaks move to lower energies as diameter increases. For all wires, P1 displays a blueshift character under increasing strain, though such shifts are less noticeable for NWs with smaller diameter. P2 on the other hand has a more complex combination of both blueshifts and redshifts. For D12, D24, and D32, P2 generally has a trend of being redshifted under both tensile and compressive strains. For D16, however, it generally has a trend of being blueshifted.

## Conclusions

First-principles DFT calculations were carried out to study the effect of strain on electronic and optical properties of CdSe NWs. The bandgap of CdSe wires generally reduces under increasing tensile strain. This is due to the fact that the conduction band moves down towards the Fermi level as tensile strain is applied. An interesting band-shift behavior was also found at the valence bands, where the top three valence bands in turns define VBM. The cause roots in the relative orientations between the bonds and strain. This band-shift behavior further also leads to sudden decrease in the effective mass of the holes and affects the corresponding optical properties of the wires as well. Our simulation results provide novel understandings and valuable insights on the strain effect of CdSe nanowires, which may also help the investigations of other CdSe nanomaterials.

## Abbreviations

ATK: Atomistix Toolkit
CBM: Conduction band minimum
CdSe: Cadmium selenide
GGA: Generalized gradient approximation
MGGA: Meta-GGA
NWs: Nanowires
SLS: Solution–liquid–solid
TB09: Tran and Blaha
VBM: Valence band maximum
WZ: Wurtzite
ZB: Zinc blende
